# Dietary diversity and its associated factors among school children in conflict affected communities of southern Ethiopia

**DOI:** 10.3389/fnut.2024.1462178

**Published:** 2025-01-03

**Authors:** Tagese Yakob, Eskinder Israel, Begidu Yakob, Mekdes Meshesha, Endale Jambo, Tadewos Utalo, Awoke Abraham

**Affiliations:** ^1^School of Public Health, College of Health Science and Medicine, Wolaita Sodo University, Sodo, Ethiopia; ^2^Division of Nutrition, Maternal and Child Health Unit, Wolaita Zone Health Department, Sodo, Ethiopia; ^3^Integrated Emergency Surgery and Obstetrics (IESO), Maternal and Child Health Unit, Bele Primary Hospital, Sodo, Ethiopia

**Keywords:** dietary diversity, nutrition, conflict affecting, school-age children, southern Ethiopia

## Abstract

**Background:**

Globally, more than 815 million estimated people worldwide suffer from malnutrition or are unable to access enough food due to malnutrition. Conflict remains the single most fundamental factor contributing to chronic malnutrition in the conflict-affected areas. Developing a healthy and balanced dietary pattern is essential for school children because their habits in this period may last longer. Therefore, the current study aimed to assess dietary adequacy level and factors associated among school children in conflicts affected communities of southern Ethiopia.

**Method and materials:**

A community-based cross-sectional study was conducted among 616 schoolchildren aged 6 to 12 years with their caregivers using a multistage sampling method. The data were collected by using a structured questionnaire. The data were collected and entered into Epi-data version 4.6.0.2 and exported to STATA software version 14 for analysis. Adequate dietary diversity was defined as the consumption of at least five food groups. Both bivariable and multivariable binary logistic regression were performed. A *p*-value less than 0.05 on multivariate logistic regression indicated a statistically significant association.

**Results:**

The overall response rate of this study was 98.5%. The median [inter quartile range (IQR)] dietary diversity score of the studies was 4(3–5) with the prevalence of inadequate dietary diversity among the school children of current study was 59.4% (95% CI, 55.17–62.99). In this study, schoolchildren with no near-health care facility (AOR = 1.95, 95% CI; 1.35–2.82), a family size greater than five (AOR = 1.44, 95% CI; 1.01–2.05), and the absence of a family home garden (AOR = 1.55, CI: 1.35–1.83) were significantly associated with low dietary diversity in school children.

**Conclusion:**

When compared with other studies, dietary diversity in the current study area was low. This emphasizes the need to encourage mothers to use family planning and implementing focused public health interventions, such school lunch programs and community gardening projects, to improve children’s nutritional outcomes and dietary diversity.

## Background

World Health Organization (WHO) has reported that one in every two school-aged children suffers from hunger (1). Globally, more than 815 million estimated people worldwide suffer from malnutrition or are unable to access enough food due to malnutrition (2). In Africa, nearly 40% (60 million) school children had faced malnutrition (3).

Malnutrition is the leading and most important factor for morbidity and mortality among children worldwide (4). School age is an important period of children’s life that needs the right attention and their diversified diet intake are more momentous (5). Children are very prone to malnutrition due to many reasons, such as low dietary intake, food insecurity within the household, infectious diseases, and inadequate health care (6).

Due to their slow and steady growth pattern before puberty, school-aged children require a wide range of nutrient-dense foods that must be gradually adjusted in term of quantity and portion size to meet their rising energy needs (7). Ensuring their health through the use of protein, fat, vitamin, and minerals can better support their overall growth and development (8). For children, optimal nutrition is crucial for child brain development as well as normal physical and cognitive development (9, 10). Less recommended diet decreases the health and physical growth and negatively affects their cognitive and social skills (6, 10, 11).

Conflict remains the single most fundamental factor contributing for chronic malnutrition in the conflict affected areas (12, 13). The community will be unable to work for the agriculture and basic livelihood when there is intermittent conflict and have gross effect on the community wellbeing including children (13, 14).

The “zero hunger” Sustainable Development Goal (SDG) targets 2.1 and 2.2 aims to “ensure that everyone has access all years round healthy, nutritious, and sufficient food” and “put an end to all forms of malnutrition, including eradicating hunger (7). In Ethiopia, Ministry of Education is committed to the promotion of quality health and nutrition for school-age children who constitute more than 25% of the total population (15), and of whom a major portion suffer from alarming levels of ill health, nutritional deficiencies and morbidity. In response to this, Ethiopia Federal Ministry of Health (FMOH) has been striving to eradicate malnutrition and enable the health and nutrition status to be improved for a significant proportion of Ethiopia’s population, promoting healthy attitudes, knowledge and behaviors throughout their lifetime among school age children through compressive national School Health and Nutrition (SHN) strategy (16). Despite various studies have assessed the dietary diversity of school age children in different countries including Ethiopia, among general communities, none have explored in conflict-affected communities (17–19). Careful assessing and identifying this will improve school children’s nutritional status and significantly improve overall child’s development. Therefore, the current study aimed to assess dietary adequacy level and factors associated among school children in conflicts affected communities of southern Ethiopia.

## Materials and methods

### Study area, period, and design

A community-based cross-sectional study was conducted from 16th June to 23rd July 2023 in Konso zones, southern region of Ethiopia. The Konso Zone is one of the 12 zones in the southern region of Ethiopia. The Konso Zone is divided into five districts (Konso zuriya, Kena, Koleme, Karat city administration and Segen Zuriya) with an estimated total population of 359,998 and a total of 43 Kebeles. It is situated 595 km away from the capital city of Ethiopia, Addis Ababa. Konso shares a border with the northwest Alle zone’s Burji zone in the east, the northeastern Amaro zone’s Gardula zone in the north, the Ari zone in the southwest, and the Borena zone of Oromia in the south (20). Mixed farming, or the production of both crops and animals, is the primary source of household income in many districts. The main cabbage tree in the area is *Moringa stenopetala*, also known as Shelaqta’an in Konso. Fresh green leaves are used in everyday meals as a vegetable that is cooked and consumed, and they can also be sold for cash in the neighborhood market. This area in Ethiopia is impacted by intercommunal conflict (21).

### Population, sample size calculation, and sampling procedure

All school-age children paired with their parents or caregivers in conflicts affecting the community of southern Ethiopia were the source population, and all school-age children were systematically selected from randomly sampled households with their parents or caregivers in the selected Keble. Only households with at least one school-age child aged between 6 and 12 years of age with their parent/caregiver’s pair were included in this study. Study participants who were sick during the previous week and who had a special ceremony on the day before data collection were excluded from the study.

The sample size was calculated based on the single population formula by Epi-data version 4.6.0.2 considering the following assumptions: P (proportion of dietary diversity practice among school-age children) = 58.3% of the studies conducted with school children in the Gurage Zone, Ethiopia (18); margin of error (*d*) = 5%; confidence level = 95%; *n* = (Za/2)^2^*p* (1−*p*)/d^2^; *n* = sample size = 373; and a multiplier design effect of 1.5 = 560. After considering a 10% non-response rate, the final sample size was 616.

Analogously, related studies (22, 23) have employed this methodology. Using a multistage simple random sampling technique, districts and kebeles within the study area were chosen. Four of the five districts—three rural districts and one district with an intentionally chosen karate city administration—were included in the study. Twelve kebeles (three in each) were chosen from these districts using a simple random sampling number. Next, in the chosen kebeles, every household was registered as a sampling frame. Ultimately, the sample size was distributed proportionally among the designated kebeles in the respective districts, and the respondents were selected through a computer-generated basic random sampling procedure.

### Study variables

The dietary diversity score (DDS) was used as the outcome variable in this study. The independent variables were sociodemographic characteristics (age, maternal status, maternal and paternal education status, mother and father occupational status, child sex, child birth order, resident, family size, household head, household security), environmental characteristics (water source, type of water treatment, availability of latrine, hand washing practice), comorbidity of children (chronic disease conditions, acute illness for the last 2 weeks), and access-related characteristics (animal in household of their family, own farm land, media, presence of home garden, access to fruit/vegetables, time to reach health care from home, and access to healthcare services).

### Operational definition

The dietary diversity score was defined as the total count of different food groups irrespective of the amount consumed by children in the 24-h period preceding the survey by 24-h dietary recall method. It was developed using the mother’s recollection of the food groups the child had consumed in the 24 h prior to the survey. A 10 food groups were reported by mothers as being consumed by the child: (1) cereals, roots and tubers; (2) Legumes/pulse; (3) Dairy products (milk, yogurt, cheese); (4) Flesh foods; (5) Eggs; (6) Vitamin-A rich fruits and vegetables; (7) Other fruits and vegetables; (8) nut and seeds; (9) meat and meat products and (10) other meat products (18, 24, 25) and each food groups response options were ‘yes, consumed’ (score 1) and ‘no, not consumed’ (score 0). These were added up to create child DDS, which range from 0 to 7. The outcome variable (diet diversity) was measured by inadequate/adequate. A child is considered having poor/inadequate/diet diversity when he/she eats less than four types of food groups per day (within 24 h) (26). Finally, those children who categorized adequate/meet the minimum/dietary diversity score were coded as “1” and those who categorized inadequate/did not meet minimum/dietary diversity score were coded as “0” for regression analysis.

Household security (hanger scale) was categorized into three levels based on the FANTA recommendation (24, 27).

Near-health care facility: at least health facility should be within 5 km from communities’ inhabitant or 1 h walking distance from living house to health facility.

School age: children aged 6–12 years were included (18).

### Data collection tools and quality control

The data were collected via face-to-face interviews using pretested validated food frequency questionnaires adapted from related literature (5, 6, 17–19, 23, 28, 29). It was collected from caregivers by allowing them to freely recall the type of food items they feed their children within the last 24 h.

The data collectors were trained before the data collection for 2 days on how to ask questions and code answers. Close supervision was provided by the investigators and the assigned supervisors. Any quest related to clarity, ambiguity, incompleteness, or misunderstanding was resolved on the day following the next day. Pretests were performed on 5% of the sample nearby city called Kelle and necessary amendments like spelling and appropriate word selection were done. The questionnaire was prepared in English, translated to the local language and checked for consistency by translating it back to English by those who were well-oriented with the stated languages. All finalized data collection forms were checked for completeness and clarity before and during data management, storage and analysis.

### Data processing and analysis

The data were entered into Epi-data version 4.6.0.2 and exported to STATA software version 14 for analysis. Before the analysis of the data, the variables were recoded to facilitate analysis. Descriptive statistics such as the frequency for all variables and median with interquartile range (IQR) some specific variable were computed. Multicollinearity regression coefficient based was checked by the help of variance inflation factor based on value of VIF <10 that updates no collinearity among independent predictors. Binary logistic regression was performed to examine the association of each independent variable with the outcome variable. Variables with *p*-value <0.25 in the bivariable analysis were selected and included in the final multivariable logistic regression analysis. Multivariate logistic regression was performed to identify the independent predictors of outcome variable. Associated variable was tested at 95% of CI and summarized by using AOR and any variable with *p*-value <0.05 in the final model was taken as statistically significant. Model fitness was checked by using Hosmer and Lemeshow’s test, with a *p*-value greater than 0.05 indicating a fitted regression model (that was *p*-value = 0.905). After checking for multi-collinearity, the associations between dependent and independent variables were measured using odds ratio (OR) with 95% confidence interval (CI) at *p*-value <0.05. Finally, the results of the study are presented in the form of text, tables, and figures.

## Results

### Sociodemographic characteristics of the respondents

The overall response rate of this study was 98.5%. Majority of households (91.1%) household head was fathers. Nearly half (45.9%) of the heads of household were aged 20–29 years. A significant percentage of mothers (72.3%) were housewives and about 58.3% had no formal education. About nearly two-third, (62.4%) of fathers were farmers/herdsmen, and greater than one-third, 255 (42.0%) of fathers had completed primary school. More than half of the school children (325, 53.5%) were male, nearly two-thirds, 370 (60.1%) of the school children were aged 6–8 years, and more than one-third, 246 (40.5%) of the children were intermediate birth order. The majority, 530 (87.3%) of study participants were living in rural areas (Table 1).

**Table 1:** Sociodemographic characteristics of respondents in conflict-affecting communities in the southern region of Ethiopia, 2023.

Variables (*n* = 607)	Category	Dietary diversity practice (%)		*p*-value
		Adequate	Inadequate	
Head of household	Fathers	226(40.9)	327(59.1)	0.986
	Caregivers	22(40.7)	32(59.3)	
Age category of head of household (year)	≤20	12(32.4)	25(67.6)	0.720
	20–29	118(42.3)	161(57.7)	
	30–39	91(40.6)	133(59.4)	
	≥40	27(40.3)	40(59.7)	
Occupational status mother	Housewife	166(37.5)	277(62.5)	<0.001
	Government Employee/NGO	23(82.1)	5(17.9)	
	Merchant	45(42.5)	61(57.5)	
	Farmer	14(46.7)	16(53.3)	
Educational status mother	No formal education	128(36.2)	226(63.8)	**<0.001**
	Primary school	60(36.8)	103(63.2)	
	Secondary school	27(49.1)	28(50.9)	
	College and above	33(94.3)	2(5.7)	
Occupation of father	Farmer/herdsmen	139(36.7)	240(63.3)	<0.001
	Merchants	62(39.5)	95(60.5)	
	Government/NGO employee	47(66.2)	24(33.8)	
Educational status of father	No formal education	86(37.2)	145(62.8)	<0.001
	Primary school	90(35.3)	165(64.7)	
	Secondary school	50(56.8)	38(43.2)	
	College and above	22(66.7)	11(33.3)	
Sex of children	Male	125(38.5)	200(61.5)	0.197
	Female	123(43.6)	159(56.4)	
Age category of children (year)	6–8	150(40.5)	220(59.5)	0.868
	9–12	94(41.2)	134(58.8)	
Birth order of children	First	89(46.1)	104(53.9)	0.154
	Middle	91(36.9)	155(63.1)	
	Last	68(40.5)	100(59.5)	
Number of school child in household	One	129(43.6)	167(56.4)	0.428
	More than one	119(36.6)	192(63.4)	
Residence	Rural	198(37.4)	332(62.6)	<0.001*
	Urban	54(66.6)	27(33.4)	
Number of family size	≤5	98(33.6)	194(66.4)	<0.001*
	>5	150(47.6)	165(52.4)	

Bold indicates significant level variable at *p*<0.05. *Significant level *p*<0.05.

### Environmental characteristics of the respondents

Greater than one-third, 241 (39.7%) of the study participants used communal water. The majority (505, 83.1%) had access to water near their residence area, and more than one-third (33.9%) of the respondents did not use any type of water treatment. Greater than two-thirds (77.3%) of the respondents had a toilet. Overall, 454 (74.8%) practiced hand washing either before or after eating or before or after food processing (Table 2).

**Table 2:** Environmental characteristics of respondents in conflict affecting communities in the southern region of Ethiopia, 2023.

Variables (*n* = 607)	Category	Dietary diversity practice (%)		*p*-value
		Adequate	Inadequate	
Water source	Protected water	38(45.2)	46(54.8)	0.763
	River water	23(36.5)	40(63.3)	
	Unprotected water	20(44.4)	25(55.6)	
	Pond	21(41.2)	30(58.8)	
	Communal	92(38.2)	149(61.8)	
	Pipe	23(32.3)	69(67.6)	
Distance of water	Near	212(41.9)	293(58.1)	0.210
	Far	36(35.3)	66(64.7)	
Treatment of water	Yes	115(41.3)	163(58.7)	0.381
	No	133(40.4)	196(59.6)	
Latrine availability	Yes	198(42.2)	271(57.8)	0.209
	No	50(36.2)	88(63.8)	
Hand washing practice	Yes	192(42.3)	262(57.7)	0.216
	No	56(36.6)	97(63.4)	
Hand washing before meal	Yes	182(39.8)	275(60.2)	0.367
	No	66(44.0)	84(56.0)	
Hand washing after meal	Yes	180(39.4)	277(60.6)	0.199
	No	68(45.4)	82(54.6)	
Hand washing after toilet	Yes	181(39.6)	276(60.4)	0.274
	No	67(44.7)	83(55.3)	
Hand washing before food processing	Yes	186(40.6)	272(59.4)	0.829
	No	62(41.6)	87(58.4)	
Hand washing before food processing	Yes	182(40.0)	273(60.0)	0.458
	No	66(43.4)	86(56.6)	

### Comorbid conditions of the school children

Of the total school children, 96 (23.6%) had chronic diseases. Overall, 78 (18.8%) of the school-aged children had fever 2 weeks prior to data collection. Of these, 25 (32.1%) had related diarrheal disease, and 24 (30.7%) had upper respiratory tract infections (Table 3).

**Table 3:** Comorbidity conditions of schoolchildren in conflict affecting communities in the South Region, Ethiopia, in 2023.

Variables	Category	Dietary diversity practice (%)		*p*-value
		Adequate	Inadequate	
Chronic disease	Yes	40(41.7)	56(58.3)	0.860
	No	208(40.7)	303(59.3)	
Acute illness last 2 weeks	Yes	25(30.9)	56(69.1)	0049
	No	223(42.4)	303(57.6)	
Types of illness	Diarrheal	4(17.4)	19(82.6)	0.434
	Acute upper tract infection	8(33.3)	16(66.7)	
	Acute febrile diseases	11(40.7)	16(59.3)	
	Other specify	2(33.2)	4(66.7)	

### Access-related characteristics of the respondents

The majority, 527 (86.8%) of study participant had animals in the household of their family, and a significant number of households, 424 (69.8%), had their own farmland. The majority, 524 (86.3%) of their households had access to any type of media. Greater than one-third, (469, 77.3%) of the participants had home gardens and nearly two-thirds, (386, 63.6%) had access to fruits/vegetables. Nearly two-third, 357 (58.8%) of the respondents were near the market to their living house, and nearly half, (323, 53.2%) had access to healthcare services within ≤1 h (Table 4).

**Table 4:** Access-related characteristics of school-aged children in conflict-affecting communities in the South Region, Ethiopia, in 2023.

Variables (*n* = 607)	Category	Dietary diversity practice (%)		*p*-value
		Adequate	Inadequate	
Presence of animal in household	Yes	196(33.2)	331(62.8)	<0.001*
	No	52(65.0)	28(35.0)	
List of animals	Chicken only	18(45.0)	22(55.0)	0.010
	Chicken, and sheep/goat	25(40.9)	36(59.1)	
	Cow, and sheep/goat	22(26.7)	66(73.3)	
	Chicken, and cow	19(26.1)	54(73.9)	
	chicken, cow, and sheep/goat	82(44.6)	102(55.4)	
	Chicken, cow, sheep/goat	30(37.1)	51(62.9)	
Presence of farm land	Yes	157(37.0)	267(63.0)	0.003
	No	91(49.7)	92(50.3)	
Harvest list	Sorghum only	8(53.4)	7(46.7)	0.821
	Sorghum and bean	14(45.2)	17(54.8)	
	Sorghum, barely	15(39.5)	23(60.5)	
	Sorghum potatoes	15(36.6)	26(63.4)	
	Sorghum, bean and barley	44(34.1)	85(65.9)	
	Sorghum, bean, and potatoes	33(40.7)	48(59.3)	
	Sorghum, barley, and potatoes	13(36.1)	23(63.9)	
	Sorghum, bean, barley, and potatoes	55(35.9)	98(64.1)	
Presence of any media	Yes	219(41.8)	305(58.2)	0.238
	No	29(34.9)	54(65.1)	
List of media exist	Radio only	132(42.8)	203(57.2)	0.003
	Radio, and Phone	34(34.0)	66(66.0)	
	TV, and Radio	58(69.9)	25(30.1)	
	TV, and phone	27(75.0)	9(25.0)	
	Radio, TV, and phone	2(50.0)	2(50.0)	
Presence of home garden	Yes	172(36.7)	297(63.3)	<0.001*
	No	138(69.0)	62(31.0)	
List of home garden product	Moringa only	22(28.2)	56(71.8)	0.094
	Cabbage only	47(44.8)	58(55.2)	
	Moringa and cabbage	26(28.8)	64(71.2)	
	Cabbage, and hurt	15(41.7)	21(58.3)	
	Moringa, and hurt	53(39.7)	35(60.3)	
	Moringa, hurt, and cabbage	45(41.7)	63(58.3)	
Access to fruit and vegetables	Yes	140(36.3)	246(63.7)	0.002
	No	108(48.9)	113(51.1)	
Presence of near market	Yes	126(5.3)	231(64.7)	0.001
	No	122(48.8)	128(51.2)	
Distance of market (kilometer)	<1	52(49.1)	54(50.9)	0.538
	1–2	55(52.4)	50(47.6)	
	≥2	15(41.7)	21(58.3)	
Presence of near-health care facility	Yes	101(31.3)	222(68.7)	<0.001
	No	147(51.8)	137(48.2)	

TV, television. *Significant level *p*<0.05.

### Household food security

About 42.3% of respondents were secured with food access scales. In the past 4 weeks, 46.7% were worried about household food shortages, 48.6% could not eat the kinds of foods they preferred, 40.3% ate a limited variety of foods, 39.2% ate some foods that truly did not want to eat, 37.5% ate a smaller meal than you felt, 46.8% ate a fewer meal a day, 36.2% went to sleep at night hungry and 33.1% went a whole day and night eating nothing (Table 5).

**Table 5:** Household food insecurity of respondents in conflict affecting communities in the South Region, Ethiopia, 2023.

Variables (*n* = 607)	Category	Dietary diversity practice (%)		*p*-value
		Adequate	Inadequate	
In the past 4 weeks, did u worry that your household would not have enough	Yes	116(41.1)	166(58.9)	<0.877
	No	132(40.6)	193(59.4)	
In the past 4 weeks, were you or any not able to eat the kinds of foods they preferred	Yes	120(40.7)	175(59.3)	0.931
	No	128(41.1)	184(58.9)	
In the past 4 weeks, did you or any household member have to eat a limited varieties	Yes	104(42.3)	142(57.7)	0.973
	No	144(39.9)	217(60.1)	
In the past 4 weeks, did you or any household member have to eat some foods truly did not want to eat	Yes	1029(42.9)	136(57.1)	0.421
	No	146(39.6)	223(60.4)	
In the past 4 weeks, did you or any household member have to eat a smaller meal than you felt	Yes	113(39.8)	171(60.2)	0.616
	No	135(41.8)	188(58.2)	
In the past 4 weeks, did you or any household member have to eat a fewer meal a day	Yes	84(39.8)	133(60.2)	0.216
	No	164(42.1)	226(57.9)	
In the past 4 weeks, did you or any household member go to sleep at night hungry	Yes	160(41.4)	227(58.6)	0.746
	No	88(40.0)	132(60.0)	
In the past 4 weeks, did you or any no food to eat of any kind in your household day-night	Yes	81(40.3)	120(59.7)	0.274
	No	167(41.2)	239(58.8)	
Overall household food insecurity	Yes	105(40.5)	154(59.5)	0.891
	No	134(39.5)	205(60.5)	

### Dietary diversity practice

**Figure 1:**
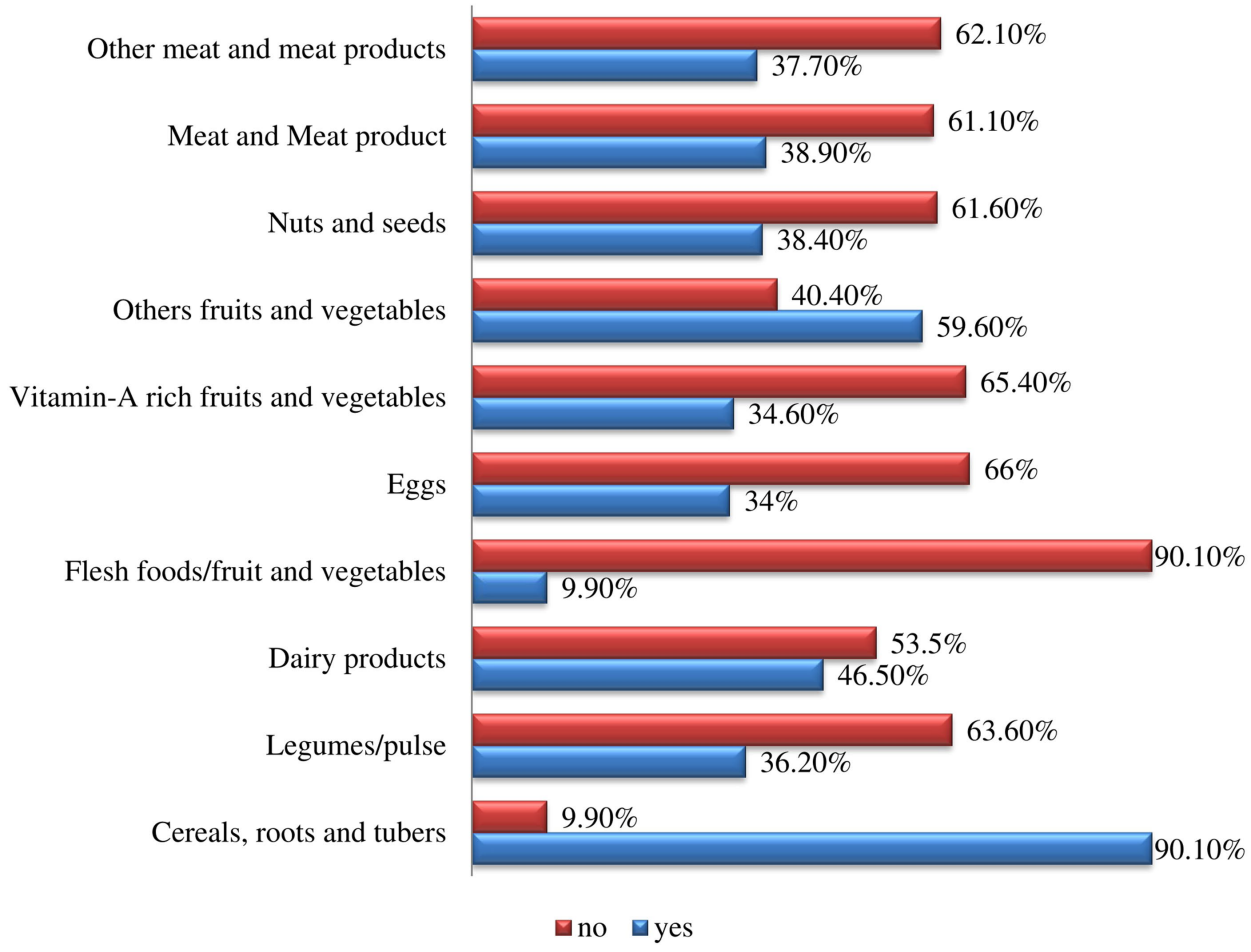
A 10 food group dietary diversity measured using the 24-h recall method for schoolchildren in conflict-affecting communities in the southern region of Ethiopia in 2023.

**Figure 2:**
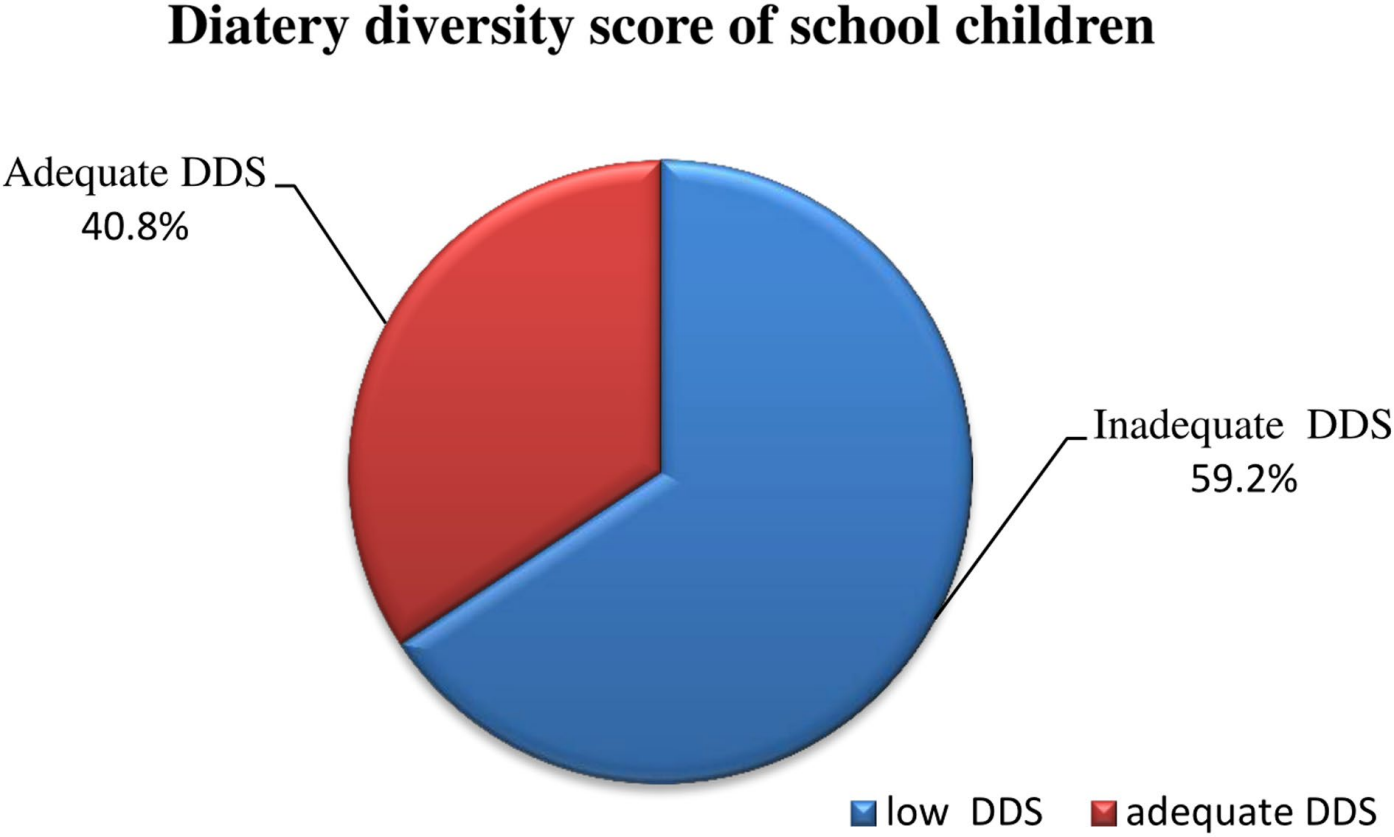
Measure of dietary diversity practices of schoolchildren in conflict affecting communities in the South Region, Ethiopia, in 2023.

The median (IQR) dietary diversity score of the participants was 4 (3–5). The prevalence of study participants with inadequate dietary diversity in this study was 59.4% (95% CI, 55.17–62.99). Flesh food (9.9%) was the food group least consumed by the school-age children, and cereals, roots and tubers (90.1%) were the most consumed food group by the school-age children (Figures 1, 2).

### Factors affecting school-age children’s dietary diversity practices

All the independent variables were analyzed via logistic regression with the dependent variable of school children’s dietary diversity practices to analyze their associations. Nine variables were significantly associated with school dietary diversity according to binary logistic regression (*p*-value ≤0.25) and were entered into the multiple logistic regression analysis. There was a significant association between the following variables: educational level of the father, residence, number of family members, birth order, time to reach health care from home, fruit/vegetable access, presence of a home garden, presence of an animal, and presence of a market resident area.

In this study, schoolchildren who were not at a health care facility were 1.95 times higher odds of having inadequate diet diversity than were their counterparts (AOR = 1.95, 95% CI; 1.35–2.82). Schoolchildren with family sizes greater than five were 1.44 times higher odds of having inadequate diet diversity than their counterparts were (AOR = 1.44, 95% CI; 1.01–2.05). School children with no family home garden were 1.55 times higher odds of having inadequate diet diversity [AOR = 1.55, CI: (1.35–2.03)], which was significantly associated with inadequate dietary diversity in the study area (*p*-value <0.05) (Table 6).

**Table 6:** Bivariable and multivariable logistic regression analysis of factors associated with school children in conflict affecting communities in the southern region of Ethiopia in 2023.

Variables	Category	Dietary diversity practice (%)		COR (95% CI)	AOR (95% CI)	*p*-value
		Adequate	Inadequate			
Educational level of father	No formal education	86(37.2)	145(62.8)	1(reference)	1(reference)	
	Primary school	90(35.3)	165(64.7)	0.92(0.63–1.33)	0.78(0.53–1.15)	0.128
	Secondary school	50(56.8)	38(43.2)	2.21(1.34–3.65)	1.59(0.92–2.76)	0.100
	College and above	22(66.7)	11(33.3)	3.37(1.55–7.29)	1.07(0.39–2.94)	0.881
Residence	Rural	198(86.4)	332(13.6)	0.29(0.17–0.47)	0.77(0.32–1.87)	0.573
	Urban	54(75.8)	27(24.2)	1(reference)	1(reference)	
Number of family size	≤5	98(33.6)	194(66.4)	1 (reference)	1 (reference)	
	>5	150(47.6)	165(52.4)	1.79(1.29–2.49)	1.44(1.01–2.05)	0.042*
Birth order of children	First	89(46.1)	104(53.9)	1 (reference)	1(reference)	
	Middle	91(36.9)	155(63.1)	0.68 (0.46–1.01)	0.71(0.47–1.08)	0.054
	Last	68(40.5)	100(59.5)	0.79(0.52–1.21)	4.38(0.51–1.23)	0.281
Access to Fruit/vegetable	Yes	140(36.3)	246(63.7)	0.59(0.42–0.83)	0.96(0.65–1.43)	0.875
	No	108(48.9)	113(51.1)	1(reference)	1(reference)	
Presence of home garden	Yes	172(88.8)	297(11.2)	1(reference)	1(reference)	
	No	138(81.8)	62(18.2)	2.11(1.44–3.11)	1.55(1.35–2.03)	0.043*
Presence of animal	Yes	196(33.2)	331(62.8)	0.31(0.19–0.52)	0.80(0.35–1.83)	0.602
	No	52(65.0)	28(35.0)	1(reference)	1(reference)	
Presence of near market	Yes	126(5.3)	231(64.7)	0.57(0.411–0.79)	0.90(0.61–1.33)	0.611
	No	122(48.8)	128(51.2)	1(reference)	1(reference)	
Presence of near-health care facility	Yes	101(31.3)	222(68.7)	1(reference)	1(reference)	
	No	147(51.8)	137(48.2)	2.35(1.69–3.28)	1.95(1.35–2.82)	**<0.001**

Hosmer and Lemeshow Test *x*^2^ = 3.419, df = 8 and *p*-value = 0.905 shows that model fit and VIF = 1.59 shows that no multicollinearity among dependent variable. Bold indicates significant level variable at *p*<0.05. *Significant level *p*<0.05.

## Discussion

This community-based study assessed dietary diversity and its predictors among school-age children in the southern region of Ethiopia. The median (IQR) dietary diversity score of the participants was 4 (3–5), and the prevalence of inadequate dietary diversity among the schoolchildren in this study was 59.4% (95% CI, 55.17–62.99). This finding was lower than that reported in a previous study (30), which was 19.2%, and a study conducted in India (31). This variation might be due to the variation of food groups included, environmental and cultural differences between study settings. The results of our study align with those of a study conducted in the Gurage Zone, southern Ethiopia (58.3%) (18). This might due to similarity socioeconomic characteristic of both study setting. However, the results of this study were better than those of studies conducted in the EDHS (10.8%) (32), Merawi town, Amhara region, Ethiopia (8%) (25), Sinan districts (13%) (33), the Gonder Zone (16.2%) (34), the Mirab Abaya district in the southern region of Ethiopia (34.3%) (35) and the Wolaita Sodo region (43.2%) (36). Possible explanations for these variations could be the sociocultural, economic, and geographical differences between the study area and the referenced places. Further, this might be because a significant number of participants in the study area had animals in the household of their family, their own farmland and access to fruit/vegetables may have affected their dietary practices compared with those in other studies.

The study revealed that children from extended families were 1.44 times higher odds of having inadequate diet diversity (AOR = 1.44, 95% CI; 1.01–2.05). A study carried out in the Gurage Zone in southern Ethiopia provided evidence in favor of this finding (18). This might be due number of family size increase related with increase demand of recommended diet and later it may lead deficit of recommended diet at household level. Additionally it explained as the equitable distribution of food among household members according to their physiological needs is essential, as one strategy for mitigating global malnutrition is making food more accessible at home. Our research revealed that a sizable average family size exists because a significant proportion of households had two or more school-age children. Given that the total fertility rate in the country is approximately 4.6 (37), ensuring equitable food distribution within households is crucial for addressing marginalized and nutritionally vulnerable populations.

According to this study, there was a 1.95-fold increase in the probability of being close to a health care facility (AOR = 1.95, 95% CI; 1.35–2.82) for every schoolchildren with diverse diets. This comparable with study conducted in Southwest Nigeria (38–40). Further, this study also supports finding from Nepal (29), which revealed that declining dietary diversity was significantly and negatively correlated with household spending and community infrastructure (hospital presence) [lowest versus highest household expenditure quintile, less developed (without hospital) versus more developed communities (29)]. This explained by that evident that people’s intention to feed varied food to their children arises when the awareness and directive of an attitude allows the person to practice those particular health behaviors. Thus, enhancing the educational level of caregivers, particularly people responsible for primary care of the child coupled with conducive environments such as physical and economical access to food, is important (41). Further, this might be explained that, if there are no access health settings and thus limited food options.

School children with no family home garden were 1.55 times higher odds of having inadequate diet diversity [AOR = 1.55, CI: (1.35–2.03)], which was significantly associated with a *p*-value <0.05 in the study area. This finding is consistent with an analogous study conducted in the South Gondar Zone, Northwest Ethiopia (23) and in Nepal, it revealed that access to home gardens was positively correlated with high dietary diversity and negatively correlated with low dietary diversity and that home gardens have high levels of species diversity as research findings suggest that rural households with home gardens have a greater probability of transitioning from a medium dietary diversity status (42). This could be because home gardeners have the ability to gather excess food from their farms to substitute for foods that are in short supply or non-existent at home. There ought to be a wide range of food available in home gardens. A varied family diet is the result of the diversity of plants in the garden (43). Furthermore, it explained as growing fruits, vegetables, and other crops at home allows you to have access to a wide range of food that might not be found in store. In both rural and urban locations, home gardens make it simple to obtain fresh vegetables and animal products (44). Strengthen of the current the study were use of large sample size and multistage sampling method for representativeness of the sample. Whereas the limitations of current study were socioeconomic variable that may be associated with dietary diversity, such as household income were not addressed and as its nature of a cross-sectional study inference of causality is not allowed. There may be recall bias related to dietary intake information, even though; great attention was given to the study procedures; including the process of training and close supervision throughout the study period.

## Conclusion

When compared with other studies, dietary diversity in the current study area was low. A lack of proximity to a health care facility, a family size greater than five, and an absence of a family home garden were determinants of inadequate dietary diversity. A health extension platform should be used to raise awareness of the issue of inadequate dietary diversity in the area, and backyard vegetation should be improved. In addition to nutritional counseling for caregivers and the household as a whole, enhanced behavioral change programs should be implemented through community health agents, agricultural extension workers, and volunteers. The encouragement and maintain healthy program with Strong stakeholders’ collaboration is compulsory to improve healthy eating and to address family planning needs. Together with the rural roads authority, local government should support the local market and trade business that facilitates the development of market linkages. Such enterprises and initiatives would potentially communities to exchange their food commodities, which may help to increase their purchasing power and their dietary diversity status. Further, to address variable not covered in current study, a longitudinal study design is advised.

## Data Availability

The raw data supporting the conclusions of this article will be made available by the authors, without undue reservation.
